# Communicative competencies anchored longitudinally – the curriculum “personal and professional development” in the model study programme in undergraduate medical education at the University of Witten/Herdecke

**DOI:** 10.3205/zma001453

**Published:** 2021-03-15

**Authors:** Claudia Kiessling, Florian Mennigen, Heike Schulte, Laura Schwarz, Gabriele Lutz

**Affiliations:** 1 Universität Witten/Herdecke, Fakultät für Gesundheit, Lehrstuhl für die Ausbildung personaler und interpersonaler Kompetenzen im Gesundheitswesen, Witten, Germany; 2 Universität Witten/Herdecke, Fakultät für Gesundheit, Department für Humanmedizin, Witten, Germany; 3 Gemeinschaftskrankenhaus Herdecke, Psychosomatische Abteilung, Herdecke, Germany

**Keywords:** communicative competencies, communication skills, longitudinal curriculum, simulated patients, Witten/Herdecke University

## Abstract

**Background: **In October 2018, the University of Witten/Herdecke (UW/H) launched the new reformed medical education programme called Medicine 2018+. A major innovation compared to the existing model programme was the introduction of thematic focuses. A longitudinal communication skills curriculum was integrated into the new thematic focus “professional and personal development – inner work” (IAP). With the start of the new programme, the IAP curriculum has been built step-by-step over time, i.e. the first four semesters have already been implemented, the following semesters are being planned.

**Project description: **IAP aims to provide students with patient-centred medicine. Five areas of competence were defined: Doctor-patient communication, team competence, staying healthy, my paths to becoming a doctor, medicine and society. The focus of this article is on the communication curriculum. The first year of study focusses on the training of basic communication skills. In the 2^nd^ year of study, students practice these basic skills in small groups with simulated patients (SP), whereby the emphasis in the 3^rd^ semester is on history taking and in the 4^th^ semester is on sharing information. In the 3^rd^ semester, students complete a communication station in an OSCE. From the 5^th^ semester onwards, the focus of training is on the consolidation of basic and advanced communication skills, which can be applied in clinical clerkships, and the reflection of clinical experiences. Key didactic elements are, in addition to teaching the theoretical basics, experience-based small group work with and without SP, feedback and reflection. The evaluation of the summer semester 2020 showed high agreement ratings of the students regarding the overall satisfaction with the individual courses (83-100% agreement).

**Discussion: **The introduction of IAP has been very positively received by the students. Challenges are the adequate handling of the increasing student workload when planning new courses, the implementation of a longitudinal e-portfolio as well as the recruitment and training of clinical teachers and SPs.

**Conclusion: **As the evaluation results of the summer semester 2020 show, the first steps of implementing a longitudinal communication curriculum at UW/H have been successful. Helpful strategies were the orientation on published examples from other faculties as well as regular feedback and discussions with students and teachers in order to adapt and integrate educational considerations into the existing model programme in Witten.

## Zusammenfassung

**Hintergrund: **Im Oktober 2018 startete die Universität Witten/Herdecke (UW/H) mit dem neuen Modellstudiengang Medizin 2018+. Eine wesentliche Neuerung im Vergleich zum bestehenden Modellstudiengang war die Einführung von Themenschwerpunkten. In den neuen Themenschwerpunkt „Berufliche Persönlichkeitsentwicklung – Innere Arbeit“ (IAP) wurde das longitudinale Kommunikations-Curriculum integriert. Mit dem Start des neuen Modellstudiengangs wächst das IAP-Curriculum auf, d.h. die ersten vier Semester wurden bereits durchgeführt, die weiteren Semester sind in Planung.

**Projektbeschreibung: **IAP hat zum Ziel, den Studierenden eine patient*innenzentrierte Medizin zu vermitteln. Fünf Kompetenzbereiche wurden definiert: Arzt*in-Patient*in Kommunikation, Teamkompetenz, Gesund bleiben!, Meine Wege hin zum Arzt/zur Ärztin sowie Medizin und Gesellschaft. Der Schwerpunkt des Artikels liegt auf der Darstellung des Kommunikations-Curriculums. Im ersten Studienjahr werden kommunikative Basiskompetenzen vermittelt. Im 2. Studienjahr üben die Studierenden diese Basiskompetenzen in kleinen Gruppen mit Simulationspatient*innen (SP), wobei im 3. Semester der Schwerpunkt auf der Anamnese und im 4. Semester auf der Weitergabe von Informationen liegt. Im 3. Semester durchlaufen die Studierenden eine Kommunikationsstation in einem OSCE. Ab dem 5. Semester liegt der Unterrichtsfokus auf der Vertiefung von Themen in Anlehnung an klinische Blöcke und der Reflexion klinischer Erfahrungen. Zentrale didaktische Elemente sind neben der Vermittlung der theoretischen Grundlagen die erfahrungsbasierte Kleingruppenarbeit mit und ohne SP, Feedback und Reflektion. Die bisherige Evaluation des Sommersemesters 2020 zeigt hohe Zustimmungswerte der Studierenden hinsichtlich der Gesamtzufriedenheit mit den einzelnen Veranstaltungen (83-100% Zustimmung).

**Diskussion: **Die Einführung von IAP ist von den Studierenden sehr positiv aufgenommen worden. Herausforderungen stellen der angemessene Umgang mit dem zunehmenden Workload der Studierenden bei der Planung neuer Veranstaltungen dar, die Implementierung eines longitudinalen e-Portfolios sowie die Rekrutierung und Schulung klinisch tätiger Dozierender und die Rekrutierung von SP.

**Fazit:** Wie die Evaluationsergebnisse des Sommersemesters 2020 zeigen, sind die ersten Schritte der Implementierung eines longitudinalen Kommunikations-Curriculums an der UW/H gelungen. Hilfreiche Prozessschritte waren dabei die Orientierung an den publizierten Beispielen anderer Fakultäten sowie die regelmäßigen Rückmeldungen und Diskussionen mit Studierenden und Dozierenden, um die Konzepte an den bestehenden Modellstudiengang in Witten anzupassen und zu integrieren.

## 1. Background

The importance of communication and social skills for the healthcare professions has been increasingly recognized in recent decades. Numerous studies have demonstrated the positive effects of successful clinical communication [1], [2], [3], [4]. From this, the demand is derived to teach communication and social skills already in undergraduate medical education. Many national catalogues of learning objectives define communicative competence as one of the key competencies that medical students must acquire during their studies [5], [6], [http://www.nklm.de], [https://www.profilesmed.ch/]. In addition to these catalogues, which describe the entire spectrum of medical competencies, specific catalogues of learning objectives have been developed that define communicative and social competencies in greater detail [7], [8], [9], [10], [11], [12]. These general and specific catalogues of learning objectives form an essential foundation for the development and implementation of longitudinal communication curricula in which, ideally, teaching, learning and assessment of communication and social skills are integrated into medical education programmes throughout the entire course of study [13], [14], [15].

The University of Witten/Herdecke (UW/H) has a long tradition of reforming medical education [16], [17]. In 1982, UW/H was the first German university to be founded under private sponsorship. In 1983, the first class of medical students began their studies in a medical programme, which has been reformed and further developed ever since. In winter semester 2018/2019, UW/H launched a new reformed medical programme, the Witten Model Programme Medicine 2018+. Essential core elements of this new course of studies were the introduction of new thematic focuses: “outpatient health care”, “professional and personal development – inner work”, “interprofessional education”, “scientific competence” as well as “tracks” in the clinical part of the study programme in the sense of a student-oriented elective curriculum. The aim of the new thematic focuses is to build on existing elements and expand them into longitudinal curricula [16], [17]. The introduction of the new study programme will take place successively, i.e. starting with the students who started their studies at the UW/H in October 2018. Important core elements of the already existing model programme, such as problem-oriented learning and the strong practical orientation with a large proportion of clinical teaching, remain unchanged in the design of the new study programme [18], [19].

In addition to the implementation of the new model curriculum, the UW/H was faced with the opportunity and challenge of doubling the number of medical students, i.e. since the summer semester 2019, 84 students per semester will begin their medical studies instead of 42 as before. 

The aim of this article is to present the conception and implementation of the longitudinal communication curriculum, which has been integrated into the thematic focus “professional and personal development – inner work” (IAP), and to report on initial experiences and challenges. 

## 2. Thematic focus “professional and personal development – inner work” (IAP)

The IAP curriculum supports students in developing the personal and interpersonal skills they will need as doctors in contact with patients and their families and in a variety of team situations in their future work. The guiding principle is patient-centred medicine, which perceives the patient and the doctor as self-determining individuals and is based on the biopsychosocial perspective on illness and health [20], [21]. 

Situated learning and reflecting on one’s own experience and actions in these learning experiences as a future doctor plays a prominent role. To support this process, several teaching formats have already been successfully implemented at UW/H in the past, for example the clinical reflection training in the practical year [22]. The aim of IAP is to promote a better understanding of one’s own and others’ needs, values and expectations, one’s own ability of reasoning and social responsibility for one another. An important aspect is also the strengthening of students’ competence to shape their professional environment in order to contribute to structural changes in the sense of patient-centred health care. The IAP curriculum comprises five fields of competence, which are trained from the first to the 10th semester:

1. doctor-patient communication (communication curriculum)
2. team competence
3. stay healthy! – Resilience and doctors’ health
4. my paths to becoming a doctor
5. medicine and society

The goals and contents are planned step-by-step each semester and so far cover the first four semesters. For the clinical phase of studies, initial concepts are oriented towards the students’ needs in the new clinical setting well as the needs of and necessities at the clinical sites. All courses are subject to continuous further development on the basis of students’ feedback and curricular conditions. Courses usually comprise a combination of theoretical input (e.g. models, study results, techniques), experience-based learning in small groups (e.g. in role-plays) and time for reflection and mutual exchange. Close links with other parts of the curriculum are actively strived for (e.g. psychosomatic medicine, problem-based learning, outpatient health care, interprofessional education, integrated studies in anthroposophical medicine). 

The article focuses on the longitudinal communication curriculum. However, the other areas of the IAP curriculum are briefly presented below, as they take up and deepen the content of the communication curriculum.

“Team competence”: This area of competence serves to strengthen the ability to work together in various team situations. It derives its necessity from the connection between teamwork quality, patient safety and emotional stability or exhaustion of doctors [23]. The goals are to promote reflection on one's own role in the team as well as the willingness and ability to support each other and to communicate successfully. A simplified version of Deci and Ryan's Self-Determination Theory provides an essential basis for a better understanding of one’s own and others' behaviour [24], [25].

**“Stay healthy!”:** This area serves to reflect and strengthen students’ resilience. It is intended to make a preventive contribution to keeping students healthy and to prevent the widespread health challenges among medical students and doctors, such as burnout [26], [27], addiction [28] or a higher suicide rate [29], [30]. At the same time, the positive effects of good communication on the stressful life of doctors are discussed [20] and ways are developed to act professionally even in challenging conversational situations. 

**“My paths to becoming a doctor”: **This series of activities is intended to promote conscious reflection and shaping of one’s own professional and personal development. In the Anglo-American world, this process is known as Professional Identity Formation (PIF), which starts at the beginning of studies, develops in interactive relationships and is influenced by the norms and expectations of others as well as by one’s own experiences, cognitions, motives and emotions [31], [32]. A central role is attributed to role models and the reflection of personal experiences in clinical and non-clinical settings. Reflection and feedback play a central role in raising awareness of these processes [31], [33], [34]. 

In the mentoring programme, which was already introduced in 2013, students have, for example, the opportunity to identify and cultivate their own values and motives, but also to learn to implement them flexibly and realistically in exchange with older students and clinical teachers [35]. The main aim of this programme is to recognise and experience the importance of personal and interpersonal reflection and openness in dealing with personal issues as these can serve as facilitating or challenging factors in developing owns own professional role.

**“Medicine and Society”: **This series of events aims to familiarise students with various medical disciplines, to discuss current health policy issues with experts and to reflect on the future of the medical professions. The topics are oriented towards the students’ interests; the events themselves are conceptualized and organized by students. In the winter semester 2019/20, an evening event on the topic of abortion and §219a [36] and a documentary film presentation with subsequent panel discussion on the topic of “the market-driven patient” took place [37], [38].

### 2.1. Doctor-patient communication

#### Learning objectives and contents

The communication curriculum takes up a large part of IAP (35.5 hours out of a total of 63 hours in the first four semesters). It is based on the learning objectives of the European Consensus on inter- and multi-professional educational objectives (HPCCC) by the Teaching Committee of the International Association for Communication in Healthcare (TEACH), which has been translated into German [39]. It can be perceived as a further development of the Basel Consensus Statement [10], [11]. The curriculum design is also based on the structure of the Calgary Cambridge Guide (CCG) [40] and the Basel curriculum “Social and communicative skills” [41]. To illustrate the theoretical framework model of the CCG, it is presented to students in each training session of the communication curriculum. In courses with simulated patients (SP), the scheme serves as an observation sheet that students use to provide feedback (see attachment 1 ).

In the pre-clinical phase, the focus is on basic communication skills: Building relationship, providing structure in a conversation, dealing with emotions, gathering information and sharing information. The aim is to train basic communication skills to students, enabling them to apply their communicative skills in the clinical phase of studies with real patients and to reflect their own strengths and areas of development. During the clinical phase, subject-specific and specifically challenging situations are taken up and the trained communication skills of the pre-clinical phase are applied and further developed, based on the contents of the clinical clerkships.

##### Didactics

Course units offer a mixture of theoretical input and practical exercises. Theoretical content includes communication models (e.g. NURSE model for dealing with emotions [42]), but also the presentation of selected studies [43], [44] and – depending on the topic – the use of videos. Experience-based learning is mainly implemented in role-plays with and without SP. As an example, the didactic sequence of the course unit “Providing structure in a conversations”, which is the first communication course in the first semester, is shown in attachment 2 . 

Training with SP takes place in so-called simulated patient contact (SPC) in the 3^rd^ and 4^th^ semester. The focus of the course lies on the application of the basic skills learned in the 1^st^ and 2^nd^ semester and on experience-based learning. Students attend three two-hour sessions in each semester. In each session, there is a short introduction followed by 2-3 medical interviews, i.e. each student conducts one interview during the semester. In the 3^rd^ semester, the focus is on the topic “history taking” or “gathering information”. The interview setting is in a family doctor's practice, at an accident insurance consultant or in an emergency room. The patients’ complaints range from inner restlessness to a bicycle accident or chest pain. The interviews last either five or eight minutes, depending on the complexity of the patient’s problem. After each interview, there is a feedback session in which first the student who conducts the interview, then the SP and then the observers provide their feedback. The way of providing feedback is based on the schemes “perception – effect – suggestion” for those giving feedback and “listen – ask back – summarise” for those receiving feedback. Following the feedback round, students can discuss open questions or ask questions to the SP. It is the task of the trainers to facilitate the process and to supplement the feedback if necessary. In the 4^th^ semester, the focus is on the topic of “sharing information”. The didactic approach is comparable to that of the 3^rd^ semester. The encounters take up topics such as discharge, information giving e.g. before an outpatient operation and risk communication (using the example of cancer screening). Specific educational activities are carried out in cooperation with other thematic focusses, e.g. with the Ambulant Health Care (AGV) or Interprofessional Education (IPE). Table 1 (Tab. 1) provides an overview of the communication curriculum in semesters 1 to 4.

##### Assessment and compulsory attendance

As a model study programme according to §41 of the Licensing Regulations, the UW/H conducts replacing licensing examinations in the first four semesters, namely three written examinations in the style of Modified Essay Questions (MEQ) in the 2^nd^, 3^rd^ and 4^th^ semesters and two OSCEs in the 2^nd^ and 3^rd^ semesters. In winter semester 2019/20, one integrated station (out of 12) was included in the examination for history taking and communication skills for the first time in the OSCE of the 3^rd^ semester. 

The philosophy of the UW/H encompasses that only very few courses are compulsory in the programme, e.g. problem-based learning, physical examination courses, Studium fundamentale. Therefore, most of the courses in the IAP curriculum are voluntary. Only the SPC in the 3^rd^ and 4^th^ semester were compulsory courses from the beginning. Attendance at voluntary IAP activities fluctuates between 10 and 60% depending on the year. For this reason, it was decided in spring 2020 to make one course unit compulsory in each of the 1^st^ and 2^nd^ semesters.

##### Organisation

The communication curriculum, together with the SP programme, is located at the chair for the training of personal and interpersonal competencies in health care (“Lehrstuhl für die Ausbildung personaler und interpersonaler Kompetenzen im Gesundheitswesen”). A working group has been set up for the conception of the IAP curriculum, which meets monthly and consists of the chair’s staff, students and representatives of the Integrated Studies in Anthroposophical Medicine (IBAM), and psychosomatic medicine. Courses are organised by the staff of the chair. Coordination with other thematic focusses are also discussed in regular meetings. Courses are carried out by the staff of the chair and by external trainers, especially clinical psychologists and medical doctors. Some courses are also held together with student co-facilitators (e.g. team competence) or are in the hands of students. In winter semester 2019/20, a total of about 200 hours of instruction took place for semesters 1-3.

##### Quality assurance and evaluation

Quality assurance measures include regular meetings and feedback rounds with teachers and students as well as discussions in the IAP working group. Since the cohorts of students at UW/H are well organised, it is also possible to obtain feedback on IAP or individual courses via the cohort representatives. An evaluation concept based on online surveys was developed with the faculty's evaluation officer for the summer semester 2020.

#### 2.2. Evaluation results in the summer semester 2020

The evaluation of IAP courses was carried out by means of an online questionnaire and comprised seven closed questions (7-point scale from 1=fully agree to 7=disagree) and two open questions. The SPC in the 3^rd^ and 4^th^ semester was evaluated separately with a more comprehensive online questionnaire (using the same 7-point scale). It should be noted that all IAP events in summer semester 2020 had to be held digitally due to the COVID-19 pandemic. At the end of June 2020, 42 completed questionnaires were available for the event “providing structure in a conversation” in the first semester (50% response rate). Overall, 95% of the students were satisfied with the event (scale points 1-3; fully agree - rather agree). In the second semester, 13 and 20 responses were received for the courses “the art of history taking” and “dealing with emotions” (responses 16% and 50%). Overall, 95% and 100% of the students were satisfied with these courses. For the SPC in the 3^rd^ semester, 23 responses were received (response rate 27%). Of these, 74% stated that they had improved their communication skills as a result of the SPC. 83% were satisfied with the SPC overall. For 87%, the SPC addressed a relevant topic. The SPC in the 4th semester was evaluated by 21 students (response rate 50%). Of these, 100% stated that they had improved their communication skills through the SPC and 94% considered the topic to be relevant. 

## 3. Discussion

The IAP courses have now been running for four semesters and have been very positively evaluated by the students. However, participation in the voluntary, non-examination-related courses is in some cases low. One reason for this is probably the conception of the model study programme 2018+, which introduced four new thematic focuses, but did not reduce any of the existing courses and content. This means that students are confronted with a rather considerable additional workload. Another reason for low participation rates are the written examinations, which are perceived by some students at the beginning of their studies to be unsettling and demanding in terms of content. Therefore, many students concentrate on courses and contents that are relevant to the exams. 

This was one of the reasons why a station on the topic of communication and history taking was added to the OSCE in the 3^rd^ semester. At the moment, there are also discussions about integrating IAP contents into the written examinations in the 2^nd^ and 4^th^ semester. This is possible because IAP includes subjects of medical psychology and medical sociology, which are part of the Physikum, the first part of the licensing examination after two years. At the same time, when planning new courses, it must always be taken into consideration not to increase the student workload even further. In other words, the IAP courses will continue to be very focused and limited in time.

“Advertising” plays a major role in increasing the number of participants in IAP events. Students receive course descriptions in order to inform themselves about the learning objectives and contents. In addition to that, one week before the course students receive a flyer as an announcement via the student messenger groups.

Within the framework of the new conception of IAP, some projects could not be implemented so far, e.g. an accompanying e-portfolio. The aim would be to offer students a platform in which they can upload e.g. reflective writing, written work, video analyses and self-recorded videos, in order to receive feedback on their work from fellow students and teachers. At the moment, the technical requirements are still lacking. However, there is hope that a longitudinal e-portfolio can be implemented in the coming semesters. 

A particular challenge will be the growing effort for teachers, not only in terms of teaching, but also in terms of providing individual feedback to students. For example, it was originally planned for the first semester to provide students with a video of a doctor-patient interview, which they were to analyse by means of processing tasks. The plan was to provide individual written feedback to all students. In the 1^st^ semester, about 50% of the students handed in such a video analysis. The effort required of the teachers to write the written feedback was about 30 minutes per student, i.e. with 84 students this would be over 40 hours of work. For capacity reasons, this is unfortunately not feasible at present.

The recruitment of external trainers, especially clinicians, also proves to be challenging. In times of increasing workload in hospitals, an enormous commitment is needed to be willing and prepared to teach comparatively new subjects such as clinical communication in addition to patient care. In other words, there is an additional need for training in the hospitals, both in terms of successful clinical communication and modern didactics. It is positive, however, that in discussions with clinicians, the topic of communication is very positively received and the need is recognised by all sides as long as teaching is oriented towards the clinical context, needs and questions. However, the implementation is a tour de force for all.

## 4. Conclusion

In Witten, we have taken the first steps towards a longitudinal communication curriculum. Many things have worked well, while others have not yet. What encourages us is the very positive feedback from students and the openness and interest of the clinicians. The next steps include the planning and implementation of the courses in the clinical phase of studies, oriented towards the contexts and questions of the clinicians and students, the recruitment and training of teachers and simulated patients, the integration of communication skills assessment into the examinations and a longitudinal e-portfolio. We were able to benefit greatly from the preliminary work of other medical faculties, e.g. Basel, Berlin, Freiburg, Leipzig and Neuruppin, for the development of the IAP curriculum. We would therefore like to take this opportunity to thank all our colleagues, also at other locations.

## Acknowledgements

We thank all current and former members of the working group IAP (Yinka Aranmolate, Friedrich Edelhäuser, Katja Frost, Patrick Giemsa, Charlotta Hülsmann, Katharina Ladermann, Lisa Lombardo, Ayam Mankiewicz, Nikolaus Munzig, Sarah Schößer, Diethard Tauschel, Andrea Witowski), all students for their constructive and critical feedback, our “comrades-in-arms” in the other thematic focusses and in the Dean's Office, as well as all participating teachers and simulated patients for their great commitment, without which our work would not be possible. We would like to thank Angelika Taetz-Harrer for providing us with the results of the evaluation.

## Profiles

**Name of the location: **Universität Witten/Herdecke

**Field of Study/ Profession: **Model course of study Medicine 2018+ in the field of study Human Medicine

**Number of learners per year or semester: **84 per semester

**Is a longitudinal communication curriculum being implemented? **In process: The first three semesters have already been implemented, further semesters are being planned/ implemented 

**In which semesters are communicative and social skills taught? **According to current plan: 1^st^-12^th^ semester

**What teaching formats are used? **Interactive seminars, practical exercises with and without simulation patients, e-learning 

**In which semesters are communicative and social skills tested (formative or pass relevant and/ or graded)? **Currently: Relevant to pass 1 OSCE station in a state exam substitute OSCE in the 3^rd^ semester.

Planned: Formative testing formats, e.g. e-portfolio, online-based self-tests

**What testing formats are used? **OSCE, e-Portfolio, written tests

**Who (e.g. clinic, organization) is in charge of development and implementation? **Chair for the education in personal and interpersonal competencies in health care 

Interested lecturers of clinical fields (e.g. psychosomatics, inner medicine, clinical psychology, psychotherapie, pediatrics)

## Current professional roles of the authors

- Claudia Kiessling studied medicine in Berlin and public health in Bielefeld. She completed her habilitation in Munich in the field of medical didactics. She is currently professor and chair at the University of Witten/Herdecke and is responsible for the topic “professional personality development” in the model course of studies in medicine. She is co-chair of the GMA committee “Communicative and social competencies”.
- Florian Mennigen studied psychology in Boston, USA and in Bochum, Germany. He is a licensed psychological psychotherapist. He currently works as a psychological psychotherapist and is a course facilitator at the University of Witten/ Herdecke in the areas of team competence and resilience in the subject area “professional and personal development” of the model course of study in medicine.
- Heike Schulte is a graduate psychologist and systemic consultant (SG). After twenty years in human resources and as an entrepreneur in management development, she now works at the University of Witten/ Herdecke as a research assistant and doctoral candidate with a focus on professional identity formation, resilience and team competence.
- Gabriele Lutz studied medicine in Witten-Herdecke. She completed her clinical and academic training in Herdecke, Cleveland and Seattle, USA. She is currently head of the Psychosomatic Department at the Community Hospital in Herdecke. Academically, she works on topics in the field of professional and personal development and the training of intra- and interpersonal competencies in medical education.

## Competing interests

The authors declare that they have no competing interests. 

## Supplementary Material

Introductory slide for training sessions on doctor-patient communication

Advance organizer of the training session "Providing structure in a medical interview" (1st semester)

## Figures and Tables

**Table 1:**
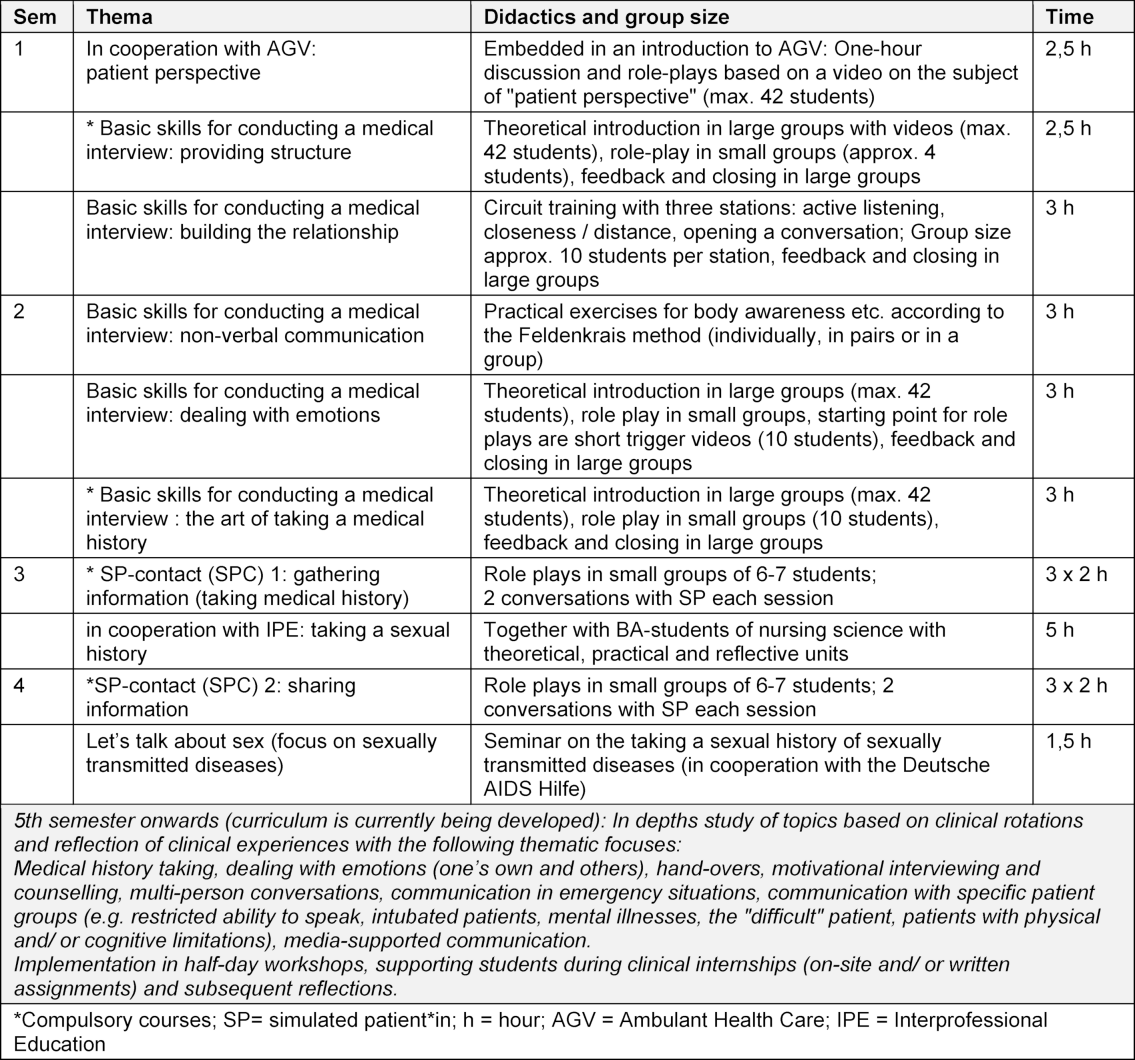
Overview of the longitudinal communication curriculum

## References

[1] Stewart MA (1995). Effective physician-patient communication and health outcomes: a review. Can Med Assoc J.

[2] Ong LM, de Haes JC, Hoos AM, Lammes FB (1995). Doctor-patient communication: a review of the literature. Soc Sci Med.

[3] Beck RS, Daughtridge R, Sloane PD (2002). Physician-patient communication in the primary care office: a systematic review. J Am Board Fam Pract.

[4] Rao JK, Anderson LA, Inui T, Frankel RM (2007). Communication Interventions Make A Difference in Conversations Between Physicians and Patients A Systematic Review of the Evidence. Med Care.

[5] Accreditation Council for Graduate Medical Education (ACGME) (2003). Outcome Project.

[6] General Medical Council (2018). Outcomes for graduates 2018.

[7] Simpson M, Buchman R, Stewart M, Maguire P, Lipkin M, Novack D, Till J (1991). Doctor-patient communication: the Toronto consensus statement. Brit Med J.

[8] Makoul G (2001). Essential elements of communication in medical encounters: the Kalamazoo consensus statement. Acad Med.

[9] Von Fragstein M, Silverman S, Cushing A, Quilligan S, Salisbury H, Wiskin C, UK Council for Clinical Communication Skills Teaching in Undergraduate Medical Education (2008). UK consensus statement on the content of communication curricula in undergraduate medical education. Med Educ.

[10] Kiessling C, Dieterich A, Fabry G, Hölzer H, Langewitz W, Mühlinghaus I, Pruskil S, Scheffer S, Schubert S (2008). Basler Consensus Statement "Kommunikative und soziale Kompetenzen im Medizinstudium": Ein Positionspapier des GMA-Ausschusses Kommunikative und soziale Kompetenzen. GMS Z Med Ausbild.

[11] Kiessling C, Dieterich A, Fabry G, Hölzer H, Langewitz W, Mühlinghaus I, Pruskil S, Scheffer S, Schubert S, Committee Communication and Social Competencies of the Association for Medical Education Gesellschaft für Medizinische Ausbildung, Basel Workshjop Participants (2010). Communication and social competencies in medical education in German-speaking countries: the Basel Consensus Statement. Results of a Delphi Survey. Pat Educ Couns.

[12] Bachmann C, Abramovitch H, Barbu CG, Cavaco AM, Elorza RD, Haak R, Loureiro E, Ratajska A, Silverman J, Winterburn S, Rosenbaum M (2013). A European consensus on learning objectives for a core communication curriculum in health care professions. Patient Educ Couns.

[13] Silverman J (2009). Teaching clinical communication: A mainstream activity or just a minority sport?. Pat Educ Couns.

[14] Bachmann C, Hölzer H, Dieterich A, Fabry G, Langewitz W, Lauber H, Ortwein H, Pruskil S, Schubert S, Sennekamp M, Simmenroth-Nayda A, Silbernagel W, Scheffer S, Kiessling C (2009). Longitudinales, bologna-kompatibles Modell-Curriculum "Kommunikative und Soziale Kompetenzen": Ergebnisse eines interdisziplinären Workshops deutschsprachiger medizinischer Fakultäten. GMS Z Med Ausbild.

[15] Kiessling C, Frischenschlager O, Hladschik-Kerner B (2013). Longitudinales Curriculum "Kommunikative und soziale Kompetenzen". Gesprächsführung in der Medizin. Lernen, lehren, prüfen.

[16] Butzlaff M, Hofmann M, Fuhrmann M, Güdler J, Kohler J, Pohlenz P, Schmidt U (2014). Der Modellstudiengang Medizin an der Universität Witten/Herdecke - auf dem Weg zur lebenslang lernfähigen Arztpersönlichkeit. Handbuch Qualität in Studium und Lehre.

[17] Frost K, EdelhäuserF, HofmannM, Tauschel D, Lutz G (2019). History and development of medical studies at the University of Witten/Herdecke- an example of "continuous reform". GMS J Med Educ.

[18] Scheffer C, Tauschel D, Neumann M, Lutz G, Valk-Draad M, Edelhäuser F (2013). Active student participation may enhance patient centeredness: patients' assessments of the clinical education ward for integrative medicine. Evid Based Complement Alternat Med.

[19] Scheffer C, Valk-Draad MP, Tauschel D, Büssing A, Humbroich K, Längler A, Zuzak T, Köster W, Edelhäuser F, Lutz G (2018). Students with an autonomous role in hospital care - patients perceptions. Med Teach.

[20] Mead N, Bower P (2000). Patient-centredness: a conceptual framework and review of the empirical literature. Social Sci Med.

[21] Kiessling C, Frischenschlager O, Hladschik-Kerner B (2013). Patientenzentrierte Kommunikation. Gesprächsführung in der Medizin. Lernen, lehren, prüfen.

[22] Lutz G, Scheffer C, Edelhaeuser F, Tauschel D, Neumann M (2013). A reflective practice intervention for professional development, reduced stress and improved patient care-A qualitative developmental evaluation. Pat Educ Couns.

[23] Welp A, Meier LL, Manser T (2016). The interplay between teamwork, clinicians' emotional exhaustion, and clinician-rated patient safety: a longitudinal study. Crit Care.

[24] Ryan RM, Deci EL (2000). Self-Determination Theory and the Facilitation of Intrinsic Motivation, Social Development, and Well-Being. Am Psychol.

[25] Deci EL, Ryan RM (2000). The "What" and "Why" of Goal Pursuits: Human Needs and the Self-Determination of Behavior. Psychol Inquiry.

[26] Bergner TM (2010). Burnout bei Ärzten.

[27] Zwack J (2013). Wie Ärzte gesund bleiben - Resilienz statt Burnout.

[28] Reimer C, Jurkat HB, Mäulen B, Stetter F (2001). Zur Problematik der Suchtgefährdung von berufstätigen Medizinern. Lebensqualität und Gesundheitsverhalten von Ärztinnen und Ärzten mit und ohne Substandabhängigkeit. Psychotherap.

[29] Reimer C, Trinkaus S, Jurkat HB (2005). Suidical tendencies of physicians - an overview. Psychiatr Prax.

[30] Hem E (2015). Suicide among doctors. Tidsskr.

[31] Goldie J (2012). The formation of professional identity in medical students: Considerations for educators. Med Teach.

[32] Cruess RL, Cruess SR, Boudreau JD, Snell L, Steinert Y (2015). A Schematic Representation of the Professional Identity Formation and Socialization of Medical Students and Residents: A Guide for Medical Educators. Acad Med.

[33] Wald HS, Anthony D, Hutchinson TA, Liben S, Smilovitch M, Donato AA (2015). Professional Identity Formation in Medical Education for Humanistic, Resilient Physicians: Pedagogic Strategies for Bridging Theory to Practice. Acad Med.

[34] Mann K, Gordon J, MacLeod A (2009). Reflection and reflective practice in health professions education: a systematic review. Adv Health Sci Educ Theory Pract.

[35] Lutz G, Pankoke N, Goldblatt H, Hofmann M, Zupanic M (2017). Enhancing medical students' reflectivity in mentoring groups for professional development - a qualitative analysis. BMC Med Educ.

[36] Szász N, Nicklaus N (2019). Am Anfang war ein "Nein".

[37] Universität Witten (2020). Der marktgerechte Patient.

[38] Franke L, Lorenz H Der marktgerechte Patient.

[39] Bachmann C, Kiessling C, Härtl A, Haak R (2016). Communication in Health Professions: A European consensus on inter- and multi-professional learning objectives in German. GMS J Med Educ.

[40] Kurtz S, Silverman J, Benson J (2003). Marrying Content and Process in Clinical Method Teaching: Enhancing the Calgary-Cambridge Guides. Acad Med.

[41] Kiessling C, Langewitz WA (2013). The longitudinal curriculum "social and communicative competencies" in the Bologna-reformed undergraduate medical education in Basel. GMS Z Med Ausbild.

[42] Langewitz WA, Edlhaimb HP, Höfner C, Koschier A, Nübling M, Leitner A (2010). Evaluation eines zweijährigen Curriculums in Psychosozialer und Psychosomatischer Medizin - Umgang mit Emotionen und patientenzentrierter Gesprächsführung. Psychother Psych Med.

[43] Mauksch LB, Dugdale DC, Dodson S, Epstein R (2008). Relationship, Communication, and Efficiency in the Medical Encounter Creating a Clinical Model From a Literature Review. Arch Intern Med.

[44] Langewitz WA, Denz M, Keller A, Kiss A, Rüttimann S, Wössmer B (2002). Spontaneous talking time at start of consultation in outpatient clinic: cohort study. BMJ.

[45] Langewitz WA (2013). DocCom.Deutsch Modul 05: Integration der patientenzentrierten und der arztzentrierten Gesprächsführung.

