# Supplementary material for: Communicative competencies anchored longitudinally – the curriculum “personal and professional development” in the model study programme in undergraduate medical education at the University of Witten/Herdecke
Source: GMS J Med Educ. 2021 Mar 15;38(3):Doc57. doi: 10.3205/zma001453 (PMC7994876; doi:10.3205/zma001453)
Supplement: Introductory slide for training sessions on doctor-patient communication [file JME-38-3-57-s-001.pdf]

Attachment 1: Introductory slide for training sessions on doctor-patient communication

After Kurtz SM, Silverman JD, Benson J, Draper J. Marrying Content and Process in Clinical Method Teaching: Enhancing the Calgary-Cambridge Guides. Academic Medicine 2003; 78(8):802-809

Feedback: Perception – Effect – Suggestion

Providing Structure

- Time-framing
- Technique like waiting, repeating, mirroring, summarising
- Techniques likes overt transitions & sign posting
- Use of questioning techniques
- Book metaphor
- Structure of a medical history

Initiating the session

- Greeting, introduction, naming of function and task
- Clarify and set the agenda
- Creation of a conversation setting that ensures confidentiality & privacy

Gathering information

- History taking
  - Acute medical history
  - Past medical history
  - Review of systems
  - Family history
  - Personal and social history
  - The patient’s perspective
- Possibly: information from relatives
- Possibly: information from others in the health care sector

Physical examination

Explanation and planning

- Information/Explanation
- Breaking bad news
- Counselling
- Shared decision-making
- Follow-up checks
- Sharing information with colleagues

Closing the session

- Forward planning
- Ensuring appropriate point of closure

Building relationship

Patient-centered communication

- Respecting the patient's agenda & autonomy
- Dealing with emotions: NURSE (naming, understanding, respecting, supporting, exploring)
- Attentive listening
- Targeted use of silence and breaks
- Appropriate use of body, language and voice

Attachment 1 to: Kiessling C, Mennigen F, Schulte H, Schwarz L, Lutz G. *Communicative competencies anchored longitudinally – the curriculum “personal and professional development” in the model study programme in undergraduate medical education at the University of Witten/Herdecke*. GMS J Med Educ. 2021;38(3):Doc57. DOI: 10.3205/zma001453
