# Supplementary material for: Communicative competencies anchored longitudinally – the curriculum “personal and professional development” in the model study programme in undergraduate medical education at the University of Witten/Herdecke
Source: GMS J Med Educ. 2021 Mar 15;38(3):Doc57. doi: 10.3205/zma001453 (PMC7994876; doi:10.3205/zma001453)
Supplement: Advance organizer of the training session "Providing structure in a medical interview" (1st semester) [file JME-38-3-57-s-002.pdf]

Attachment 2: Advance organizer of the training session "Providing structure in a medical interview" (1st semester)

Time (in min.)	Topic	Implementation
15‘	Introduction of the topic	Orientation: Calgary Cambridge Guide<br>(see attachment 1)<br>Relevance: [43, 44]: relevance of providing structure for time efficiency
40‘	Techniques and examples on videos: announcing, repeating, summarizing, paraphrasing, questioning techniques, setting time frames	4 short trigger videos with and without errors, followed by analysis, discussion in plenary session and collecting suggestions for improvement
20‘	In-depth video analysis (worked example)	Video Mr. Schragen [45]: View video, then individual work with transcript: analysing the techniques used by the doctor; collect answers and discuss in plenary session
15‘	*Break*	
45‘	Practicing the techniques in role plays	In small groups of 4 students: patient, student, 2 observers with observation tasks, followed by feedback, possibly 2nd role play
15‘	Open questions, summary, evaluation	Plenary<br>White board

Attachment 2 to: Kiessling C, Mennigen F, Schulte H, Schwarz L, Lutz G. *Communicative competencies anchored longitudinally – the curriculum “personal and professional development” in the model study programme in undergraduate medical education at the University of Witten/Herdecke*. GMS J Med Educ. 2021;38(3):Doc57. DOI: 10.3205/zma001453
